# Supplementary material for: Dynalign II: common secondary structure prediction for RNA homologs with domain insertions
Source: Nucleic Acids Res. 2014 Nov 21;42(22):13939–48. doi: 10.1093/nar/gku1172 (PMC4267632; doi:10.1093/nar/gku1172)
Supplement: SUPPLEMENTARY DATA [file supp_gku1172_nar-02021-z-2014-File012.zip › manual/RNA_class/html/class_hybrid_r_n_a.html]

RNAstructure Classes: HybridRNA Class Reference

- Main Page
- Classes
- Files

- Class List
- Class Hierarchy
- Class Members

# HybridRNA Class Reference

HybridRNA Class.
More...

`#include <HybridRNA.h>`

Inherits RNA.

List of all members.

|  |  |
| --- | --- |
|  |  |
| Public Member Functions | |
|  | HybridRNA (const char sequence1[], const char sequence2[], const bool IsRNA=true) |
|  | Constructors. |
|  | HybridRNA (const char filename1[], const int type1, const char filename2[], const int type2, const bool IsRNA=true) |
| int | FoldBimolecular (const float percent, const int maximumstructures, const int window, const char savefile[]="", const int maxinternalloopsize=30) |
|  | Predict the lowest free energy secondary structure and generate suboptimal structures using a heuristic. |
| int | FoldDuplex (const float percent, const int maximumstructures, const int window, const int maxinternalloopsize=30) |
|  | Predict the lowest free energy secondary structure for two strands that cannot form intramolecular pairs and generate suboptimal structures using a heuristic. |
| int | PartitionFunctionBimolecular (const char savefile[]="") |
|  | Predict the bimolecular partition function for a sequence (with no intramolecular pairs). |
| RNA \* | GetRNA1 () |
| RNA \* | GetRNA2 () |
| int | GetErrorCode () |
|  | Return an error code, where a return of zero is no error. |
| char \* | GetErrorMessage (const int error) |
|  | Return error messages based on code from GetErrorCode and other error codes. |
| bool | GetForbidIntramolecular () |
|  | Get whether intramolecular pairs are allowed. |
| void | SetForbidIntramolecular (const bool forbid) |
|  | Set whether intramolecular pairs are allowed. |
| void | SetProgress (TProgressDialog &Progress) |
| void | StopProgress () |
|  | ~HybridRNA () |
| Private Member Functions | |
| void | commonconstructor () |
| void | SetupBimolecular () |
| Private Attributes | |
| bool | forbidunimolecular |
| TwoRNA \* | sequences |

---

## Detailed Description

HybridRNA Class.

The HybridRNA class provides an entry point for all the bimolecular structure prediction routines of RNAstructure. The class is inherited from the RNA class and contains an instance of TwoRNA, which itself contains two instances to the class RNA. 

---

## Constructor & Destructor Documentation

|  |  |  |  |  |
| --- | --- | --- | --- | --- |
| HybridRNA::HybridRNA | ( | const char | *sequence1*[], |  |
|  |  | const char | *sequence2*[], |  |
|  |  | const bool | *IsRNA* = `true` |  |
|  | ) |  |  |  |

Constructors.

The two constuctors are available to reach the TwoRNA constructors, from which this class is built. Please see the TwoRNA constructor entries for documentation.

|  |  |  |  |  |
| --- | --- | --- | --- | --- |
| HybridRNA::HybridRNA | ( | const char | *filename1*[], |  |
|  |  | const int | *type1*, |  |
|  |  | const char | *filename2*[], |  |
|  |  | const int | *type2*, |  |
|  |  | const bool | *IsRNA* = `true` |  |
|  | ) |  |  |  |

|  |  |  |  |  |
| --- | --- | --- | --- | --- |
| HybridRNA::~HybridRNA | ( |  | ) |  |

---

## Member Function Documentation

|  |  |  |  |  |
| --- | --- | --- | --- | --- |
| void HybridRNA::commonconstructor | ( |  | ) | `[private]` |

|  |  |  |  |  |
| --- | --- | --- | --- | --- |
| int HybridRNA::FoldBimolecular | ( | const float | *percent*, |  |
|  |  | const int | *maximumstructures*, |  |
|  |  | const int | *window*, |  |
|  |  | const char | *savefile*[] = `""`, |  |
|  |  | const int | *maxinternalloopsize* = `30` |  |
|  | ) |  |  |  |

Predict the lowest free energy secondary structure and generate suboptimal structures using a heuristic.

This function predicts the lowest free energy structure and suboptimal structures. If the temperature has not been specified using the RNA base class SetTemperature and no free energies have been calculated, the thermodynamic parameters have not been read and therefore they will be read by this function call. The parameter files should be located in the directory specified by the environment variable $DATAPATH of the pwd. In case of error, the function returns a non-zero that can be parsed by GetErrorMessage() or GetErrorMessageString().

**Parameters:**
:   |  |  |  |
    | --- | --- | --- |
    |  | *percent* | is the maximum % difference in free energy in suboptimal structures from the lowest free energy structure. |
    |  | *maximumstructures* | is the maximum number of suboptimal structures to generate. |
    |  | *window* | is a parameter that specifies how different the suboptimal structures should be from each other (0=no restriction and larger integers require structures to be more different). |
    |  | *savefile* | is c string containing a file path and name for a savefile (.sav)that can be used to generate energy dot plots and to refold the secondary structure using different suboptimal structure parameters. The default is "", which results in no save file written. |
    |  | *maxinternalloopsize* | is the maximum number of unpaired nucleotides in bulge and internal loops. This is used to accelerate the prediction speed. The default is 30. |

**Returns:**
:   An int that indicates an error code (0 = no error, 5 = error reading thermodynamic parameter files, 14 = traceback error).

|  |  |  |  |  |
| --- | --- | --- | --- | --- |
| int HybridRNA::FoldDuplex | ( | const float | *percent*, |  |
|  |  | const int | *maximumstructures*, |  |
|  |  | const int | *window*, |  |
|  |  | const int | *maxinternalloopsize* = `30` |  |
|  | ) |  |  |  |

Predict the lowest free energy secondary structure for two strands that cannot form intramolecular pairs and generate suboptimal structures using a heuristic.

This function predicts the lowest free energy bimolecular structure and suboptimal structures. This function does not allow any folding constraints. If the temperature has not been specified using SetTemperature and no free energies have been calculated, the thermodynamic parameters have not been read and therefore they will be read by this function call. The parameter files should be located in the directory specified by the environment variable $DATAPATH of the pwd. In case of error, the function returns a non-zero that can be parsed by GetErrorMessage() or GetErrorMessageString().

**Parameters:**
:   |  |  |  |
    | --- | --- | --- |
    |  | *percent* | is the maximum % difference in free energy in suboptimal structures from the lowest free energy structure. |
    |  | *maximumstructures* | is the maximum number of suboptimal structures to generate. |
    |  | *window* | is a parameter that specifies how different the suboptimal structures should be from each other (0=no restriction and larger integers require structures to be more different). |
    |  | *maxinternalloopsize* | is the maximum number of unpaired nucleotides in bulge and internal loops. This is used to accelerate the prediction speed. The default is 30. |

**Returns:**
:   An int that indicates an error code (0 = no error, 5 = error reading thermodynamic parameter files, 14 = traceback error).

|  |  |  |  |  |
| --- | --- | --- | --- | --- |
| int HybridRNA::GetErrorCode | ( |  | ) |  |

Return an error code, where a return of zero is no error.

This function returns and error flag that is generated during construction by RNA(const char &filename, const int type, const bool IsRNA=true) or from CalculateFreeEnergy(). An error of zero is always no error. Other codes are errors and a c-string can be fetched for the error with GetErrorMessage().

**Returns:**
:   An integer that provides the error code.

Reimplemented from RNA.

|  |  |  |  |  |  |
| --- | --- | --- | --- | --- | --- |
| char \* HybridRNA::GetErrorMessage | ( | const int | *error* | ) |  |

Return error messages based on code from GetErrorCode and other error codes.

0 = no error <100 = Error to be fetched from RNA base class. 100-999 = Error associated with bimolecular folding, to be handled here. >=1000 = Errors for underlying sequence, get message from TwoRNA base class.

**Parameters:**
:   |  |  |  |
    | --- | --- | --- |
    |  | *error* | is the integer error code provided by GetErrorCode(). |

**Returns:**
:   A pointer to a c string that provides an error message or from other functions that return integer error codes.

Reimplemented from RNA.

|  |  |  |  |  |
| --- | --- | --- | --- | --- |
| bool HybridRNA::GetForbidIntramolecular | ( |  | ) |  |

Get whether intramolecular pairs are allowed.

**Returns:**
:   A bool that indicates whether intramolecular pairs are forbidden (true = forbidden, false = not).

|  |  |  |  |  |
| --- | --- | --- | --- | --- |
| RNA \* HybridRNA::GetRNA1 | ( |  | ) |  |

Access the underlying RNA class from an instance of TwoRNA. This is provided for use with two sequence methods. Generally, there is no need for end users to use this function.

**Returns:**
:   A pointer to the underlying RNA class for sequence 1.

|  |  |  |  |  |
| --- | --- | --- | --- | --- |
| RNA \* HybridRNA::GetRNA2 | ( |  | ) |  |

Access the underlying RNA class from an instance of TwoRNA. This is provided for use with two sequence methods. Generally, there is no need for end users to use this function.

**Returns:**
:   A pointer to the underlying RNA class for sequence 2.

|  |  |  |  |  |  |
| --- | --- | --- | --- | --- | --- |
| int HybridRNA::PartitionFunctionBimolecular | ( | const char | *savefile*[] = `""` | ) |  |

Predict the bimolecular partition function for a sequence (with no intramolecular pairs).

This function must be called to predict base pair probabilities, perform stochastic traceback, or for maximizing expected accuracy. This predicts the partition function without intramolecular pairs. If the temperature has not been specified using SetTemperature and no free energies have been calculated, the thermodynamic parameters have not been read and therefore they will be read by this function call. The parameter files should be located in the directory specified by the environment variable $DATAPATH of the pwd. In case of error, the function returns a non-zero that can be parsed by GetErrorMessage() or GetErrorMessageString().

**Parameters:**
:   |  |  |  |
    | --- | --- | --- |
    |  | *savefile* | is a c string that contains the path and filename for creating a save file. This defaults to "", which indicates no file is to be written. |

**Returns:**
:   An int that indicates an error code (0 = no error, 5 = error reading thermodynamic parameter files).

|  |  |  |  |  |  |
| --- | --- | --- | --- | --- | --- |
| void HybridRNA::SetForbidIntramolecular | ( | const bool | *forbid* | ) |  |

Set whether intramolecular pairs are allowed.

If true is passed to this function, intramolecular pairs will be forbidden in FoldBimolecular.

**Parameters:**
:   |  |  |  |
    | --- | --- | --- |
    |  | *forbid* | is a bool that indicates whether intramolecular pairs are forbid. |

|  |  |  |  |  |  |
| --- | --- | --- | --- | --- | --- |
| void HybridRNA::SetProgress | ( | TProgressDialog & | *Progress* | ) |  |

Provide a TProgressDialog for following calculation progress. A TProgressDialog class has a public function void update(int percent) that indicates the progress of a long calculation.

**Parameters:**
:   |  |  |  |
    | --- | --- | --- |
    |  | *Progress* | is a TProgressDialog class. |

Reimplemented from RNA.

|  |  |  |  |  |
| --- | --- | --- | --- | --- |
| void HybridRNA::SetupBimolecular | ( |  | ) | `[private]` |

|  |  |  |  |  |
| --- | --- | --- | --- | --- |
| void HybridRNA::StopProgress | ( |  | ) |  |

Provide a means to stop using a TProgressDialog. StopProgress tells the RNA class to no longer follow progress. This should be called if the TProgressDialog is deleted, so that this class does not make reference to it.

Reimplemented from RNA.

---

## Member Data Documentation

bool HybridRNA::forbidunimolecular `[private]`

TwoRNA\* HybridRNA::sequences `[private]`

---

The documentation for this class was generated from the following files:

- F:/RNAstructure-co-from-repository/RNAstructure/RNA\_class/HybridRNA.h- F:/RNAstructure-co-from-repository/RNAstructure/RNA\_class/HybridRNA.cpp

---

Generated on Tue Jul 30 10:52:29 2013 for RNAstructure Classes by 
 1.5.7.1
